# Social and Physical Contexts of Long-Term Services and Supports

**DOI:** 10.1093/geroni/igaa057.2501

**Published:** 2020-12-16

**Authors:** Chanee Fabius, Philippa Clarke

## Abstract

In the coming years, inevitably growing numbers of older populations will yield more older Americans with extensive medical and long-term care needs. This will lead to an increasing need for long-term services and supports (LTSS) to assist older adults with routine daily activities (e.g., bathing, dressing, medication management). There is a growing interest in understanding how social and physical environments contribute to health outcomes and the provision of services and resources for older persons with disabilities requiring assistance from LTSS. Decisions about care and subsequent experiences are likely a result of factors that extend beyond personal preference or individual factors, such as neighborhood quality, housing context, and living situations (i.e., homebound status) among community-dwelling older adults. Given population aging and the shift of LTSS from nursing homes toward community settings, there is a pressing need for more information about contextual factors that might help better develop supports for vulnerable older adults. This symposium will feature four presentations that provide novel insight regarding social and physical contextual factors contributing to LTSS. Presentations leverage data from the National Health and Aging Trends Study (NHATS), a nationally representative survey of Medicare beneficiaries aged 65 and older, and will describe: 1) associations between individual and home environment risk-factors, neighborhood-level social deprivation, and falls; 2) the relationship between neighborhood-level social deprivation and caregiving intensity (number of hours of caregiving per week) among community-dwelling older adults; 3) associations between living in single-family vs. multi-unit housing and social networks; and 4) community tenure among homebound older adults.

